# Non-catheter simple noninvasive bladder draining method with no costs

**DOI:** 10.4103/0970-1591.65409

**Published:** 2010

**Authors:** M. G. Hocking

**Affiliations:** Materials Department, Imperial College, London, UK

**Keywords:** Crede, diverticulum, no catheter, TURP, urine retention, without catheter

## Abstract

**Objectives:**

To develop a simple, rapid (8 min) non-invasive non-catheter method for draining urine from the bladder, with no introduction of infection. This is of interest to men with incomplete or no bladder emptying, and also to those with a large diverticulum. There are no running costs. It could also be cautiously explored for use by neo-bladder patients and for use in various conditions of poor detrusor function.

**Materials and Methods:**

This method is based on postural drainage used in physiotherapy. A carefully graded application of pressure, kneeling, with torso horizontal, facing downwards, supported by a 12-inch square stool-top, gave passive low-pressure voiding. If the abdominal contents approximate to a non-elastic viscous fluid, such pressure is transmitted uniformly everywhere (isostatic) and so will be equal both outside and inside the bladder, and, both outside and inside the ureters connected to it. Even if this assumption is not made, calculations show that the pressure is normally less than would cause upper tract damage. Starting with a low force was important for avoiding any upper tract damage (ureter dilation, and possible refluxing back into the kidney). Initially, a partial pre-emptying by normal urination was done (if feasible). A final stage employed a simple plastic crescent shape. Website:www.ebbflow.org.uk/Page_12x.htm.

**Results:**

Average residual bladder volumes were 43 mL.

**Conclusions:**

The method was tested for four years on one patient with low-pressure chronic retention and found successful: no complications, infections, or adverse effects.

## INTRODUCTION

A new bladder drainage method is described, with major advantages: no catheters required, no discomfort, no risk of introduction of infection, requires no special equipment, no running costs, rapid, self-applied, passive (requires no muscle exertion). These are of special relevance to India where many patients may not afford self-catheters (over 700 per year needed) and antibiotics.

The method described here may be considered by urologists for anyone who has difficulty emptying their bladder if not caused by an obstruction, who presently relies on the Crede maneuver or on self-catheterization. It must not be used to overcome an obstructed urethra (e.g. caused by an enlarged prostate or by a defective urethral sphincter or a stricture).

In this single-patient case report, no complications, no infections nor adverse effects were ever experienced over the four-year study. Frequent ultrasound scans showed no urinary stones and annual blood tests showed renal function to remain completely normal. Frequent ultrasound studies throughout the whole four-year test period showed that there were no enlarged ureters and a preexisting diverticulum did not enlarge. The detrusor hypofunction and the large diverticulum were caused by chronic urine retention prior to prostatectomy (TURP).

The aim is to give passive low-pressure voiding by a carefully graded application of pressure. Starting with a low force is important for avoiding any upper tract damage (ureter dilation, and possible refluxing back into the kidney). Thus, a pre-emptying of whatever volume is feasible by a normal urination (if feasible), or by moderate hand pressure, was done first. Urine volume voided is much increased if an egestion (bowel emptying) was done just beforehand.

## MATERIALS AND METHODS

**Figure 1:**
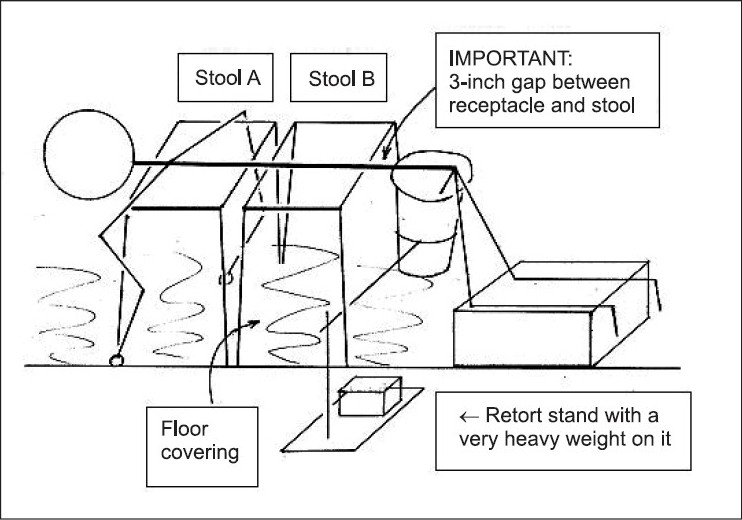
Schematic diagram: Stage 1 kneeling, using 2 kitchen stools about 18 inches high, or one miniature table; The 1-liter plastic beaker is held in a steel ring, next, for stage 2i, the 3-inch gap is closed down to zero.

A simple noninvasive method is described for draining the bladder and partially draining any diverticulum present. This method is based on postural drainage used in physiotherapy but is not mentioned in any of the standard texts on Urology (as at 2005). Method (b) required only two flat-top kitchen stools, or a miniature table. See Figure 1 for Stage 1.

A 1-liter plastic beaker was required, of about 4½ inches top diameter.

**Figure 2:**
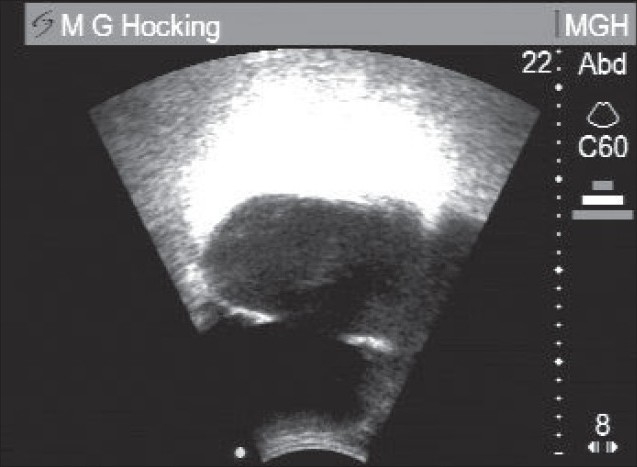
Ultrasound scan before the noninvasive drainage procedure, A large (preexisting) diverticulum is behind the bladder, Vertical dots are 1 cm apart (whole scale length is 22 cm), Before Step 1 of drainage procedure, bladder volume using V = ½ (abc) was 350 ml, after a “normal” urination

**Figure 3:**
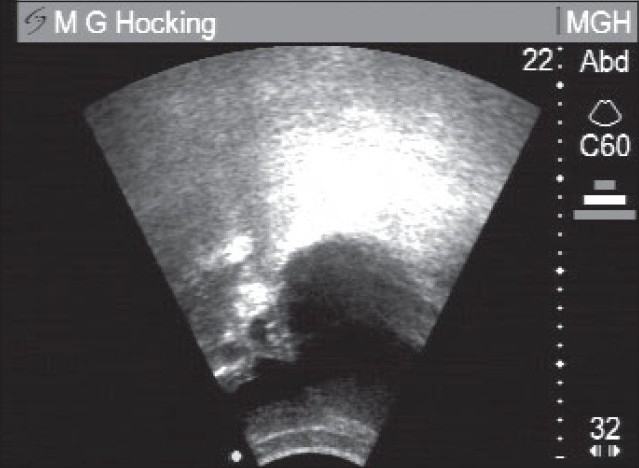
After Step 3 of the noninvasive draining procedure; Note the bladder is much smaller. Bladder is black zone near green marker dot, Approximate bladder volume V = ½ (abc) was 60 ml after the complete procedure

For Stage 2, the three-inch gap was closed. For Stage 3 a rubber crescent shape was used. Figure 2 shows the bladder region after the essential preliminary “normal” urination, but before starting on the main drainage procedure. Figure 3 shows the final result after the whole procedure.

Voided volumes are quite variable because they depend on when and how much fluid was drunk, what diet was eaten (e.g. % fruit in it), what volume of food was eaten and when, the time elapsed since the last urination and since the last egestion (bowel emptying), how full the bowel is, and how much fluid is lost in other ways (in sweat and in breath, depending on climate and activity). Such factors are not easily controllable in this type of study. For these reasons, not much significance should be placed on the standard deviation values in Column 1 of Table 1. The Column 2 values are more insulated from these factors.

**Table 1:** Total volumes voided and residual bladder volumes

Total urine volume in mL, measured by graduated receptacle	Residual bladder volume in mL measured by ultrasound
850	64
800	24
650	45
670	44
700	21
950	40
730	79
800	45
780	31
600	38
Average = 753	Average = 43
Standard deviation = 312	Standard deviation = 18

To reduce some of the above factors and to obtain some uniformity, the following regime was followed for this Table:

The measurements were taken (on consecutive days) as soon as the patient got up in the morning, instead of later during the day. No preliminary “normal” urination was done (for the purpose of these measurements only).

After Stage 1 of the method, an egestion was done (but no urination with it) before resuming the next stages. The patient normally eats a high-fruit-content diet (which helps this method) and in the previous evenings a banana was eaten to ensure that this egestion was feasible. {Note: Egestion just before (or during, by interrupting) the method gives more complete, and therefore more uniform, bladder emptying than if the bowel is full or part full.}

Table 1 was produced with the patient totally relaxed – i.e. not trying to empty the bladder by contracting the detrusor muscle.

The total early morning voided volumes above are rather high due to the subject having slept (habitually) in a cold room, which increases urine volume.

